# Hemodynamics of transcatheter tricuspid valve replacement with Lux-Valve

**DOI:** 10.3389/fcvm.2022.1007888

**Published:** 2022-10-14

**Authors:** Wang Wei, Li Ning, Ning Xiaoping, Xu Zhiyun, Li Bailing, Cai Chengliang, Yang Fan, Zhou Guangwei, Bai Yifan, Han Lin, Qiao Fan, Lu Fanglin

**Affiliations:** ^1^Department of Cardiovascular Surgery, Changhai Hospital, Naval Military Medical University, Shanghai, China; ^2^Department of Cardiothoracic Surgery, Naval Medical Center of PLA, Shanghai, China

**Keywords:** hemodynamics, tricuspid regurgitation, transcatheter tricuspid valve intervention (TTVI), LuX-Valve, transcatheter tricuspid valve replacement

## Abstract

**Objective:**

Transcatheter tricuspid valve intervention (TTVI) has emerged as an alternative treatment option for high-risk and inoperable patients with symptomatic tricuspid regurgitation (TR). However, scarce data in hemodynamic profiles were available on TTVI. In this paper, we attempt to report the hemodynamic profiles of LuX-Valve.

**Methods:**

30 patients from July 2020 to July 2021 were enrolled in this study. The patient was diagnosed with severe symptomatic TR. The clinical, invasive hemodynamic, and echocardiographic data were collected.

**Results:**

The surgical success rate was 100%. The cardiac index and stroke volume increased sharply from 2.42(2.27, 2.85) and 47.8(43.6, 62.0) to 3.04 ± 0.63 and 57.2 ± 14.7, respectively. With the elimination of TR and the increase of forward blood flow of the tricuspid valve, the extravascular lung water [798.0 (673.0, 1147.0) vs. 850.3 ± 376.1, *P* < 0.01] increased subsequently. The peak right atrium pressure decreased after Lux-Valve implantation (21.0 ± 6.4 vs. 19.4 ± 6.5, *P* < 0.05). On the contrary, the nadir right atrium pressure increased [10.0(8.0, 15.0) vs. 12.0(10.0, 17.0), *P* < 0.01]. Notably, the right atrium pressure difference dropped sharply from 9.0(5.0, 13.0) to 5.0(4.0, 8.0) after Lux-Valve implantation. There was no significant change in the pulmonary artery pressure. The right atrium volume decreased from 128(83, 188) to 91(67, 167) mL at 1 month and 107(66,157) mL at 6 months. With the remolding of the right heart chamber, the tricuspid annulus diameter shrank significantly from 42.5 ± 5.6 to 36.6 ± 6.3 mm at 1 month and 36.0 (33.0, 38.0) at 6 months.

**Conclusion:**

Invasive right atrium pressure may act as a potential candidate for TR evaluation and procedural guidance. Elimination of TR by LuX-Valve implantation improves the cardiac output and right atrium pressure and has no significant effect on the pulmonary artery pressure even with the increment of forward blood flow, suggesting the hemodynamic superiority of transcatheter tricuspid valve replacement but needs further study.

## What is known?

Compared with conservative medical treatment alone, transcatheter tricuspid valve intervention can significantly reduce the risk of rehospitalization and mortality due to heart failure.

The cardiac function and exercise tolerance were significantly improved during follow-up in severe TR patients after LuX-Valve implantation procedure, suggesting that LuX-Valve system was safe and effective in symptomatic severe TR patients.

## What the study adds?

Elimination of TR by LuX-Valve implantation improves the cardiac output and has no significant effect on the pulmonary artery pressure even with the increment of forward blood flow.

The decreased tricuspid annulus diameter and right atrium volume suggest the remolding of right heart after TR elimination.

Invasive right atrium pressure is an important parameter in hemodynamics.

## Introduction

Tricuspid regurgitation (TR) is the most common and neglected valvular heart disease of the right heart system. Secondary TR with the characteristics of right heart enlargement and tricuspid annulus (TA) dilation, which arises as a consequence of pulmonary hypertension induced by left-heart valve surgery and atrial fibrillation, takes the predominant position (1). The majority of TR patients are with the manifestation of chronic hepatic and renal insufficiency, coagulation dysfunction, and poor nutritional status on account of long-term right ventricular dysfunction (2). Hence, the mortality and complication risks of redo tricuspid valve surgery are high (3, 4). It is worth mentioning that most patients received diuretic therapy, but the symptoms of cardiac failure were not well controlled. Compared with conservative medical treatment alone, transcatheter tricuspid valve intervention (TTVI) can significantly reduce the risk of rehospitalization and mortality due to heart failure, suggesting the importance of TTVI (5). Based on the aforementioned characteristics, the 2021 European Society of Cardiology guidelines for valvular heart disease for the first time recommends TTVI as a treatment option for severe symptomatic TR patients at IIb level C (6).

TTVI is in its infancy but with a booming tendency, and gradually becomes an alternative option to minimally invasive surgery (7–9). Nowadays, TTVI includes leaflet repair, valvuloplasty, heterotopic valve replacement, and orthotropic valve replacement. The approaches include transjugular, transfemoral, and right atrium. However, the concerns for TTVI complications, including low implantation success rate, damage to the surrounding structures of the tricuspid valve (right coronary artery and conduction bundle), and device migration, have been reported in previous studies cohort. Transcatheter tricuspid valve replacement (TTVR) has captured our attention for its merit of eliminating TR instead of degradation of TR. Nevertheless, TTVR is challenging from a technical perspective. At first, the TA is a 3D shape with little calcification, which is insufficient to provide a reliable anchoring zone (10). Secondly, the diameter of the TA changes dynamically with the cardiac cycle, leading to an incomplete fit of the bioprosthesis and the native TA, which may lead to paravalvular leakage. At last, most of the currently reported orthotopic TTVR devices are based on the principle of radial force-dependent, the size of bioprosthesis is unavailable once TA is excessively dilated.

Notably, invasive hemodynamic monitoring has been the cornerstone of surgical management of valvular heart disease. With the popularity of echocardiography, the application of invasive hemodynamic monitors was once limited. However, invasive hemodynamics have been revived with the rise of TTVI recently (11). Exploring hemodynamic changes could not only guide TTVI patient selection and predict patient prognosis, but also deepen the understanding of the pathophysiology of valvular heart disease (12). Previous studies have confirmed that TR elimination after Lux-valve implantation could improve the clinical symptoms, cardiac function, and exercise tolerance of patients (13). However, scarce data in hemodynamic profiles were available on TTVI. In this paper, we attempt to report the hemodynamic changes of LuX-Valve.

## Methods

### Design and patient enrollment

A total of consecutive 30 patients (11 males) between July 2020 and July 2021 with severe TR were enrolled in this prospective study. All patients who underwent TTVR were with informed consent. The patients were comprehensively evaluated by a multidisciplinary team before surgery and deemed unsuitable for open-heart surgery. The exclusion criteria were listed below: Patients with severe pulmonary hypertension (≥55 mmHg), low left ventricular function (left ventricular ejection fraction <50%), low right ventricular function (tricuspid annular plane systolic excursion (TAPSE) <10 mm or right ventricle fractional area change (FAC) <20%), untreated severe coronary heart disease, coagulation dysfunction, and life expectancy <12 months.

The design of LuX-Valve has been described accurately previously, including a tri-leaflet bioprosthesis, ventricular septal anchor “tongue,” two leaflet-grasping clips, and an atrial disc (13–15). Preoperative evaluation of the degree of TR, hemodynamics, and right heart anatomy were achieved by echocardiography, right heart catheterization, and gated cardiac contrast-enhanced CT. Because of the complexity of the anatomical structure of the tricuspid valve complex, preoperative imaging analysis is a key factor for successful implantation. The optimal projection angle and bioprosthesis size were determined by analyzing CT before surgery. The invasive pressure of pulmonary artery, right atrium, and right ventricular were recorded before and after Lux-Valve implantation. The echocardiography data at baseline, 1 month after discharge, and 6 months after discharge were required to collect for all enrolled patients.

### Operative procedure

TTVR was performed under general anesthesia in the digital subtraction angiography operating room, and transesophageal echocardiography (TEE) was prepared in advance. The pulse indicator continuous cardiac output (PICCO, PULSION, Germany) was monitored by catheterization of the internal jugular vein and femoral artery. The cardiac output was calculated by thermodilution. The right atrial incision was adopted for the surgical approach. Double-layer 4–0 Prolene purse string suture was used for assisting the implantation of the delivery sheath. TEE was used to guide the implantation of the delivery sheath and LuX-Valve positioning during the operation.

**Figure 1:**
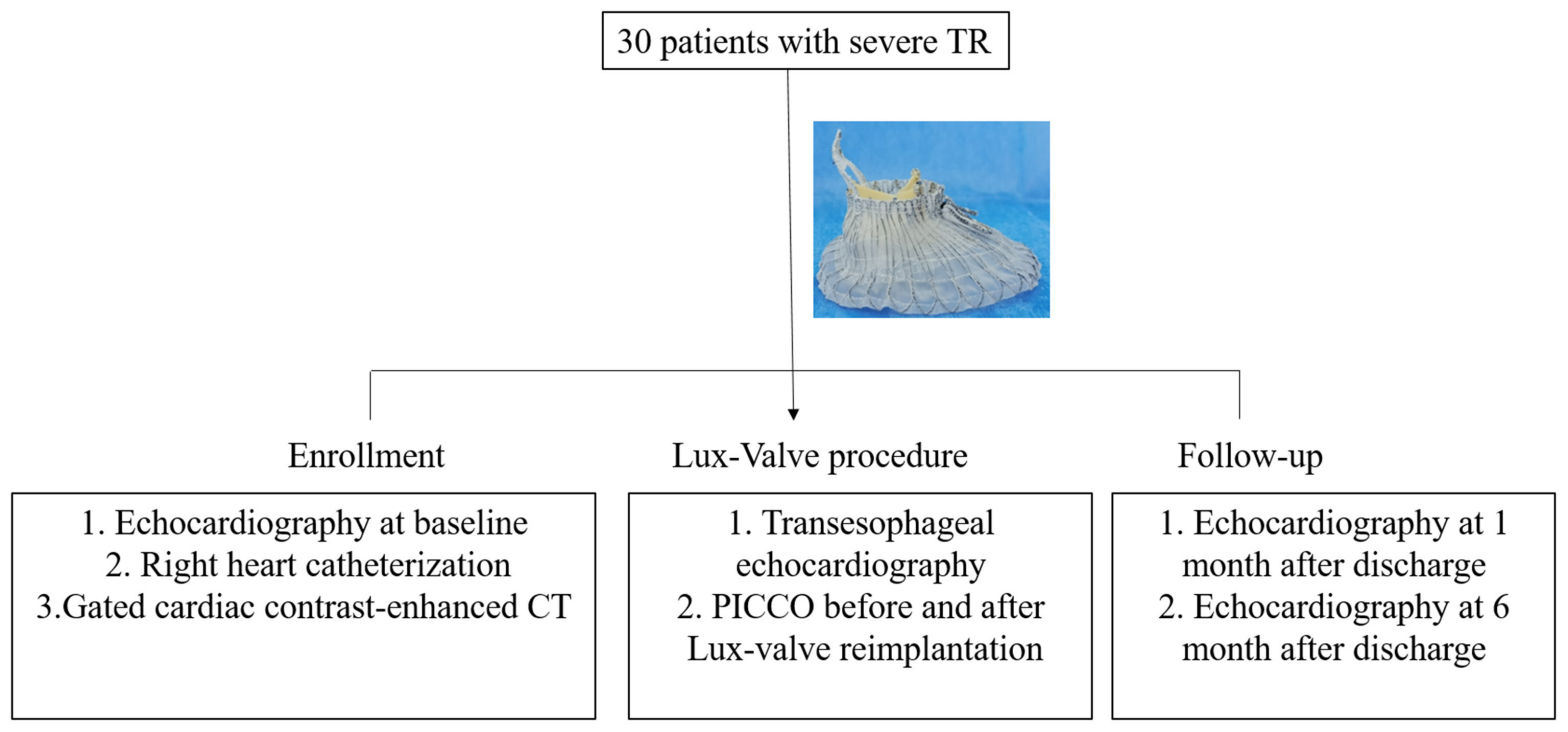
The study design for hemodynamics management of Lux-Valve.

Given the unique anchoring method of LuX-Valve and the periodic changes of the TA with the cardiac cycle, there was no requirement for strict alignment between TA and bioprosthesis plane from our experience. The delivery sheath was adjusted under the guidance of fluoroscopy and TEE to ensure its parallel direction to the interventricular septum for facilitating the fixation of the interventricular septum anchoring component. The bioprosthesis was slowly released with the retrieval of the delivery sheath. The periodical shake of grasping clips could be observed under fluoroscopy once the tricuspid anterior leaflet was hooked. And then, the atrial disc was gradually rebounded. Finally, the ventricular septal anchor “tongue” was secured to the anchoring zone. The time interval from the entry of the delivery sheath into the right atrium and the withdrawal of the sheath out of the right atrium was defined as the device time. As for the TR patients with prior permanent pacemaker implantation, the pacing lead was placed between the bioprosthesis and the native TA after Lux-Valve reimplantation. The hemodynamics was measured immediately before and after Lux-Valve implantation by PICCO. The study design for hemodynamics management of Lux-Valve was shown in Figure 1. Dopamine was used when necessary for inotropic support after surgery. Anticoagulation of warfarin was resumed once pleural fluid drainage was reduced after surgery, and low-molecular-weight heparin bridging anticoagulation was not used in this study. The average time of initiation of warfarin anticoagulation was 2.0 days post-operation. Of note, optimization of intravascular volume was performed during the perioperative period and follow-up.

## Statistical analysis

Statistical analysis was performed using SPSS V21.0 (Chicago, Illinois, USA). Normally distributed continuous variables were expressed as mean ± standard deviation, and non-normally distributed continuous variables were expressed as median (interquartile range). Categorical variables are expressed as frequencies (proportions). The comparison of normally distributed continuous variables was tested with a paired-sample *t*-test or two-way ANOVA properly. The comparison of non-normally distributed continuous variables was tested with Wilcoxon or Friedman test properly. *P* < 0.05 was considered statistically significant.

## Results

### Baseline

Table 1 presented the baseline data of the TR patients with a mean age of 65.2 years. The Society of Thoracic Surgeons (STS) and CRS scores were 8.966 ± 4.968 and 8.0 (8.0, 8.2). Kansas City Cardiomyopathy Questionnaire (KCCQ) and 6-min walk distance (6MWD) were 49.0 ± 13.0 and 259.3 ± 70.3 m, respectively. 20 patients were classified as NYHA class III. The main complaint of the patients was chest distress in 28 cases, peripheral edema in 23 cases, and abdominal distension with anorexia in 18 cases. Twenty seven patients received regular oral diuretics before surgery, 24 patients had previous left heart valve replacement surgery, including 13 cases of mitral valve replacement, 11 cases of double valve replacement. Five patients received previous permanent pacemaker implantation. The results of preoperative outcomes were shown in Table 2. The average brain natriuretic peptide was 164.9 pg/mL. The electrocardiogram result indicated that 25 patients were with atrial fibrillation. Ascites was identified by abdominal ultrasound in 3 cases. Echocardiography showed all patients were with severe TR with an instantaneous regurgitation volume of 51.7 ± 27.4 mL. The tricuspid annular plane systolic excursion (TAPSE) and fractional area change (FAC) were 15.0(13.0, 18.0) mm and 46.5 ± 6.8%, respectively. The diameter of the TA was 42.5 ± 5.6 mm (Range: 31–55 mm). Right heart catheterization result indicated that the pulmonary systolic and diastolic blood pressures were 40.2 ± 7.6 and 18.7 ± 5.1 mmHg, respectively.

**Table 1:** Patients' profile.

	**Patient (*N* = 30)**
Male	11(36.7%)
Age /years	65.2 ± 7.9
Height/cm	161.6 ± 6.7
Weight/kg	57.9 ± 8.9
Body surface area	1.62 ± 0.13
STS score	8.966 ± 4.968
CRS score	8.0(8.0, 8.2)
KCCQ score	49.0 ± 13.0
NYHA class	
III	20(66.7%)
IV	10(33.3%)
6MWD/m	259.3 ± 70.3
Symptoms	
Chest distress	28(93.3%)
Peripheral edema	23(76.7%)
Abdominal distention	18(60.0%)
Comorbidities	
Hypertension	6(20.0%)
Diabetes mellitus	3(10.0%)
Coronary artery disease	4(13.3%)
Permanent pacemaker implantation	5(16.7%)
Cerebrovascular accident	3(10.0%)
Prior surgery	
MVR	13(43.3%)
DVR	11(36.7%)
PCI	1(3.3%)
CABG	1(3.3%)
Medication	
ACEI/ARB	3(10.0%)
Beta blocker	4(13.3%)
Calcium channel blocker	2(6.7%)
Diuretic	27(90.0%)

6MWD, 6 Min Walk Distance; MVR, Mitral valve replacement; DVR, Double valve replacement; PCI, Percutaneous Coronary Intervention; CABG, Coronary Artery Bypass Grafting; ACEI, Angiotensin Converting Enzyme Inhibitor; ARB, Angiotensin Receptor Blocker.

**Table 2:** Preoperative outcomes.

	**Patient (*N* = 30)**
Laboratory test	
Leukocyte	4.75(3.57,5.99)
Hemoglobin (g/L)	122.0(106.0,133.0)
Platelet	144.3 ± 40.8
Creatinine (μmol/L)	77.0 ± 25.1
Total bilirubin(μmol/L)	20.0 ± 9.1
Direct bilirubin(μmol/L)	6.8(4.9, 10.1)
Albumin(g/L)	41.3 ± 3.6
Brain natriuretic peptide(pg/mL)	164.9(103.2, 307.5)
Electrocardiography	
Atrial fibrillation	25(83.3%)
Pacing rhythm	5(16.7%)
Ascites	3(10.0%)
Pleural effusion	0(0%)
Echocardiography	
Left ventricular ejection fraction (%)	62.9 ± 8.8
Transient Tricuspid regurgitation Volume (mL)	51.7 ± 27.4
TAPSE (mm)	15.0(13.0,18.0)
FAC (%)	46.5 ± 6.8
Tricuspid annulus (mm)	42.5 ± 5.6
Flow reversal in the inferior vena cava	30(100%)
Right heart catheterization	
Pulmonary artery systolic pressure/mmHg	40.2 ± 7.6
Pulmonary artery diastolic pressure/mmHg	18.7 ± 5.1

TAPSE, Tricuspid Annular Plane Systolic Excursion; FAC, Fractional Area Change.

### Perioperative outcome

The perioperative outcome was shown in Table 3. Surgical success was achieved in all patients. The average operation time and device time were 180.0 (140.0, 180.0) and 11.5 ± 4.4 min, respectively. The main valve size used in this study was 28–50 mm. Two cases underwent secondary thoracotomy for hemostasis due to excessive pleural fluid drainage. Additionally, 1 patient underwent redo surgery 10 days after LuX-Valve implantation on account of valve migration and died perioperatively. One patient had moderate paravalvular leakage after valve implantation. There was no occurrence of hemodialysis, new-onset of permanent pacemaker implantation, and prolonged ventilation. The ICU time and in-hospital time were 2.0 (2.0, 2.0) and 24.5 ± 7.8 days, respectively.

**Table 3:** Perioperative outcome.

	**Patient (*N* = 30)**
Operation time/min	180.0(140.0,180.0)
Device time/min	11.5 ± 4.4
Lux–valve size	
28–40	4(13.3%)
28–50	10(33.3%)
28–55	4(13.3%)
30–40	2(6.7%)
30–50	5(16.7%)
30–55	5(16.7%)
Postoperative 24h chest drainage volume (mL)	95.0(40.0,210.0)
Complications	
Hemodialysis	0(0%)
IABP	0(0%)
Permanent pacemaker implantation	0(0%)
Prolonged Tracheal Intubation (>72h)	0(0%)
Reoperation for bleeding	2(6.7%)
Reoperation for valve migration	1(3.3%)
Paravalvular leakage	1(3.3%)
ICU time/day	2.0(2.0,2.0)
In–hospital time/day	24.5 ± 7.8
Death	1(3.3%)

IABP, Intra Aortic Balloon Pump; ICU, Intensive Care Unit.

### Hemodynamic study

Of note, we monitored the hemodynamics during operation by PICCO, and the results were shown in Table 4. The cardiac index and stroke volume increased sharply from 2.42(2.27, 2.85) and 47.8(43.6, 62.0) to 3.04 ± 0.63 and 57.2 ± 14.7, respectively. The stroke index and global ejection fraction after LuX-Valve implantation were significantly higher than before operation. With the elimination of TR and the increase of forward blood flow of the tricuspid valve, the extravascular lung water increased subsequently. The extravascular lung water index and pulmonary vascular permeability index decreased significantly from 16.3 ± 6.7 and 2.7 (1.7, 3.5) to 13.0 (9.0, 20.5) and 2.0 (1.3, 3.4), respectively. In contrast, there was no significant change in the pressure of systemic circulation and pulmonary circulation.

**Table 4:** PICCO results.

	**Before TTVR**	**After TTVR**
Central venous pressure	14.9 ± 4.5	14.8 ± 4.7
Heart rate	82.1 ± 11.8	87.7 ± 11.5*
Systolic blood pressure	116.3 ± 13.9	126.5 ± 22.4*
Diastolic blood pressure	63.5 ± 12.1	66.6 ± 13.2
Cardiac index	2.42(2.27,2.85)	3.04 ± 0.63***
Stroke Volume	47.8(43.6,62.0)	57.2 ± 14.7**
SVR	1297.0(1025.5,1670.5)	1245.0 ± 376.6
SI	29.4(26.7,35.5)	33.5(30.6,38.4) ***
GEF	14.4 ± 4.0	15.0 ± 3.9*
SVV	23.0 (19.5,25.8)	17.7 ± 8.9
EVLW	798.0 (673.0,1147.0)	850.3 ± 376.1 **
GEDI	906.0 (759.0, 1030.0)	928.0 (866.5, 1016.0) *
EVLWI	16.3 ± 6.7	13.0 (9.0,20.5) **
PVPI	2.7 (1.7, 3.5)	2.0 (1.3, 3.4) **
PAPS	41.1 ± 7.5	42.6 ± 8.4
PAPD	20.0 ± 4.7	20.8 ± 4.3

PICCO, pulse indicator continuous cardiac output;TTVR, transcather tricuspid valve replacement; SVR, systemic vascular resistance; GEF, global ejection fraction; SVV, Stroke Volumee Variation; SI, stroke index; EVLW, extravascular lung water; EVLWI, extravascular lung water index; GEDI, global enddiastolic index; PVPI, Pulmonary Vascular Permeability Index.

PAPS, systolic pulmonary artery pressure; PAPD, Diastolic pulmonary artery pressure. *P < 0.05; **P < 0.01; ***P < 0.001.

In addition, the invasive pressure of right atrium and right ventricle were recorded during operation (Table 5). The peak right atrium pressure decreased after Lux-Valve implantation (21.0 ± 6.4 vs. 19.4 ± 6.5, *P* < 0.05). On the contrary, the nadir right atrium pressure increased [10.0(8.0, 15.0) vs. 12.0(10.0, 17.0), P < 0.01]. Notably, the RA pressure difference dropped sharply from 9.0(5.0, 13.0) to 5.0(4.0, 8.0) after Lux-Valve implantation. The volume of right atrium decreased from 128(83,188) mL to 91(67,167) mL at 1 month and 107(66,157) mL at 6 months after elimination of TR and the remodeling of the right heart during follow-up (Table 6). The volume of left ventricle increased significantly with the increment of forward blood flow. The TAPSE decreased significantly from 15.0(13.0, 18.5) to 10.5 ± 3.2 mm after 1 month and 11.0 ± 3.3 mm after 6 months. The FAC and LVEF decreased slightly but without significance. With the remolding of the right heart, the TA diameter shrank significantly from 42.5 ± 5.6 to 36.6 ± 6.3mm at 1 month and 36.0 (33.0, 38.0) at 6 months.

**Table 5:** Catheterization results.

	**Before implantation**	**After implantation**
Peak RV pressure	41.5 ± 7.7	44.0 ± 9.2
Nadir RV pressure	9.4 ± 7.1	7.0 ± 5.1*
Mean RV pressure	21.9 ± 5.4	22.1 ± 5.0
Peak RA pressure	21.0 ± 6.4	19.4 ± 6.5*
Nadir RA pressure	10.0(8.0,15.0)	12.0(10.0,17.0)**
Mean RA pressure	15.0 ± 4.9	15.4 ± 5.4
RA pressure difference	9.0(5.0,13.0)	5.0(4.0,8.0)**
Systolic PAP	41.4 ± 7.2	42.7 ± 8.4
Diastolic PAP	19.8 ± 5.0	20.3 ± 5.2
Mean PAP	28.0(24.0,32.0)	28.8 ± 6.0

RA, right atrium; RV, right ventricle pressure; PAP, pulmonary artery pressure. *P < 0.05; **P < 0.01.

**Table 6:** Echocardiography results.

	**Before operation**	**1 month after TTVR**	**6 months after TTVR**
RA volume/mL	128(83,188)	91(67,167)*	107(66,157)*
RV volume/mL	66(45,94)	58.6 ± 21.0	52(41,64)
LA volume/mL	133(106,213)	147(104,203)	151(107,218)
LV volume/mL	90.9 ± 23.8	104.8 ± 32.0**	103.3 ± 32.0*
Tricuspid annulus /mm	42.5 ± 5.6	36.6 ± 6.3 ***	36.0(33.0,38.0) ***
TAPSE/mm	15.0(13.0,18.0)	10.5 ± 3.2***	11.0 ± 3.3***
FAC/%	46.5 ± 6.8	45.1(40.8,48.1)	43.2 ± 11.8
LVEF/%	62.9 ± 8.8	57.0(53.5,66.0)	57.6 ± 10.3

RA, right atrium; RV, right ventricle; LA, left atrium; LV, left ventricle. TAPSE, Tricuspid Annular Plane Systolic Excursion; FAC, Fractional Area Change; LVEF, Left ventricular ejection fraction. *P < 0.05 compared to before operation; **P < 0.01 compared to before operation; ***P < 0.001 compared to before operation.

## Discussion

In this paper, we reported the hemodynamic profiles of LuX-Valve implantation. Elimination of TR by LuX-Valve implantation improves the cardiac output and has no significant effect on the pulmonary artery pressure even with the increment of forwarding blood flow. Our previous studies have demonstrated that TTVR using LuX-Valve system was safe and effective in symptomatic severe TR patients. The cardiac function and exercise tolerance were significantly improved during follow-up (13). Moreover, we, for the first time, proved the feasibility of elimination of TR by Lux-Valve instead of degradation of TR from the hemodynamic aspect.

Prolonged TR leads to hemodynamic abnormalities were verified to be associated with congestive hepatopathy and kidney dysfunction, which was associated with decreased forward cardiac output and circulation perfusion, as well as increased right-sided filling pressure and venous congestion. TR reduction by TTVR device was demonstrated to improve liver function (16). Once TR is eliminated, right atrial pressure should theoretically drop significantly. Instantaneous right atrial pressure changes combined with a marked increase in cardiac output were observed in our study, which may contribute to improved organ function and increased exercise tolerance. Further, intraprocedural invasive right atrial pressures were demonstrated to be associated with TR severity and patient outcomes after transcatheter tricuspid edge-to-edge repair. A lower RA pressure difference was proved with improved outcomes (17). Dannenberg V and colleagues assumed hemodynamic assessment before TTVR was a significant factor for patient prognosis, the logistic regression analysis verified a significant relationship between mean RA pressure and ≥1 grade TR reduction (18). Collectively, a more comprehensive investigation of invasive right atrial pressures may be needed in larger tricuspid TTVI cohorts.

It is also worth noting that in another study of transcatheter tricuspid valve-in-valve therapy for bioprosthetic valve failure, pulmonary artery pressure was increased after valve replacement. In this study, however, pulmonary artery pressure was not significantly changed. This finding led us to focus on hemodynamic studies of Lux-valve. Our results suggest that Lux-valve implantation directly eliminates TR and the increased forward flow does not result in a significant elevation of the pulmonary artery. However, with the increase of forward blood flow, it will inevitably lead to an increase in pulmonary perfusion and left ventricular preload. Therefore, the left ventricular function of TR patients must be in a normal range. For patients with abnormal left ventricular function, the choice of TTVR should be prudent. Additionally, a strengthened diuretic therapy for optimization of intravascular volume was necessary after TTVR since extravascular lung water was elevated as evidenced by PICCO.

As for the significant increase in cardiac stroke volume, it could be explained from the Frank-Starling relationship. Right ventricular stroke volume rise on account of the increase of right ventricular preload. However, a sudden increase in right ventricular filling pressure can lead to decreased compliance of the right ventricular with chronic low right ventricular preload. This may explain the reason why the right ventricular systolic function (TAPSE and FAC) decreased after surgery. Long-term chronic right and left ventricular adaptations after surgery may lead to improved postoperative exercise capacity of TR patients (19). Previous studies have also confirmed that different TTVI devices could significantly improve the clinical symptoms of TR patients, which was demonstrated by the NYHA class, 6MWD, and KCCQ score (20). Additionally, right ventricular remodeling during follow-up, including the decreased TA and the increased TAPSE and FAC level, was verified by echocardiography in previous study (21). However, a decline in TAPSE level was observed in the majority of patients during follow-up in our study. Previous studies have shown that TAPSE has no significant effect on the prognosis of TTVI patients (22). On the contrary, the patient's exercise tolerance and NYHA class during follow-up were significantly improved compared with before operation, which further suggested that TAPSE may not be suitable for assessing right ventricular function. A novel parameter may be needed for right ventricular function assessment in the future.

The application of TTVR devices was in a backward position when compared to other transcatheter repair devices. TTVR may be applicable to a broader indication because of incomplete degrees of TR reduction and functional improvement of the repair devices. In 2017, the GATE bioprosthesis (NaviGate, California, USA) was first reported for clinical application, which was implanted through the transatrial access with 100% technical success, 20% reoperation, and 20% mortality (23, 24). In addition, the EVOQUE system was also used in a compassionate cohort including 25 patients with a technical success rate of 92%. There was no occurrence of intraprocedural mortality, coronary injury, and valve migration, combined with a 100% TR level decrease (25).

TR induced by pacemaker lead could not be neglected in TTVI study, in addition, the TR patients with prior permanent pacemaker implantation were not uncommon. In this study, 5 (16.7%) of 30 cases were with prior permanent pacemaker implantation. The pacing lead was placed between the bioprosthesis and the native TA after Lux-Valve reimplantation. The risk of paravalvular leakage was low on account of the design of atrial disc. Anderson JH et al. identified that the TTVR in the setting of trans-tricuspid valve pacemaker leads without lead extraction or re-replacement can be performed safely with a low risk for complications after analyzing the data from the Valve-in-Valve International Database including 329 cases (26). Similarly, Taramasso M and colleagues verified that TTVI is feasible in selected patients with cardiac implantable electronic device and acute procedural success and short-term clinical outcomes are comparable to those observed in patients without a trans-tricuspid valve lead by analyzing the data from the TriValve registry (27).

In our previous work, we have reported the results of a compassionate multicenter study of Lux-Valve that enrolled 46 TR cases. The surgical success rate was 97.8% with 13.0% in-hospital mortality and 15.2% residual TR (13). 6 cases of 12-month follow-up data after LuX-Valve implantation have also been reported by Sun Z and colleagues (15). Of note, the incidence of paravalvular leakage in this study was 3.3%, which was lower than our previous study, further suggesting the importance of the learning curve in TTVR and emphasizing the importance of a more comprehensive understanding of tricuspid valve anatomy, hemodynamics, and surgical imaging guidance. At last, there are several limitation for our study. At first, the cardiac output was calculated by the thermodilution method in this study, which may lead to underestimation in the presence of significant TR. Secondly, this study was limited by its small cases.

## Conclusions

In summary, this study demonstrates that elimination of TR by LuX-Valve implantation improves the cardiac output and right atrium pressure instantaneously and has no significant effect on the pulmonary artery pressure even with the increment of forward blood flow. Additionally, the decreased tricuspid annulus diameter and right atrium volume further verifies the long-term remodeling of right heart after TR elimination. A more comprehensive investigation of invasive right atrial pressures may be needed in larger TTVI cohorts.

## Data availability statement

The original contributions presented in the study are included in the article/supplementary material, further inquiries can be directed to the corresponding authors.

## Ethics statement

The studies involving human participants were reviewed and approved by Shanghai Changhai Hospital Ethics Committee. The patients/participants provided their written informed consent to participate in this study.

## Author contributions

Experiment design: QF and LF. Data collection, analysis, and paper draft: LN, WW, and NX. Experiment: XZ, LB, CC, YF, BY, HL, QF, LF, and ZG. Paper revision: LF, QF, and HL. Funding: LF and LN. All authors contributed to the article and approved the submitted version.

## Funding

This study was funded by National Natural Science Foundation of China (Grant Nos. 82170376 and 82100383), 234 Panfeng project of Changhai Hospital (Grant No. 2019YXK031), and 2025 Science and Technology Innovation project of Ningbo (Grant No. 2018B10092).

## Conflict of interest

The authors declare that the research was conducted in the absence of any commercial or financial relationships that could be construed as a potential conflict of interest.

## Publisher's note

All claims expressed in this article are solely those of the authors and do not necessarily represent those of their affiliated organizations, or those of the publisher, the editors and the reviewers. Any product that may be evaluated in this article, or claim that may be made by its manufacturer, is not guaranteed or endorsed by the publisher.

## Abbreviations

RA: right atrium
RV: right ventricle
TTVI: transcatheter tricuspid valve intervention
TTVR: transcatheter tricuspid valve replacement
TR: tricuspid regurgitation
TA: tricuspid annulus
PICCO: pulse indicator continuous cardiac output.
